# Significance thresholds for the assessment of contaminated groundwater: perfluorinated and polyfluorinated chemicals

**DOI:** 10.1186/s12302-018-0142-4

**Published:** 2018-06-07

**Authors:** Karl Theo von der Trenck, Rainer Konietzka, Annegret Biegel-Engler, Jan Brodsky, Andrea Hädicke, Arnold Quadflieg, Rudolf Stockerl, Thorsten Stahl

**Affiliations:** 1 LUBW-State Institute for the Environment of the Federal State of Baden-Wuerttemberg, Griesbachstr. 1-3, 76185 Karlsruhe, Germany; 2 Present Address: Birkenweg 33, 69469 Weinheim, Germany; 3 German Environment Agency, Wörlitzer Platz 1, 06844 Dessau-Roßlau, Germany; 4 Hessian Agency for Nature Conservation, Environment and Geology, Rheingaustr. 186, 65203 Wiesbaden, Germany; 5 Department 32: Soil Protection, Contaminated Sites, Ecotoxicology, North Rhine Westphalian State Agency for Nature, Environment and Consumer Protection, Wallneyer Str. 6, 45133 Essen, Germany; 6 Hessian Ministry for Environment, Climate Protection, Agriculture and Consumer Protection, Mainzer Str. 80, 65189 Wiesbaden, Germany; 7 Bavarian Environment Agency, Bürgermeister-Ulrich-Str. 160, 86179 Augsburg, Germany; 8 Hessian State Laboratory, Am Versuchsfeld 11, 34128 Kassel, Germany

**Keywords:** Chemical risk assessment, PFAS, PFC, Groundwater, Thresholds

## Abstract

**Background:**

Per- and polyfluorinated chemicals (PFC) do not occur naturally in the environment and are, therefore, of anthropogenic origin. As a consequence of their wide range of everyday applications and their extreme persistence in the environment, PFC have become ubiquitous in nature and can, therefore, be detected in groundwater as well as in many other environmental matrices. The German States’ Water and Soil Consortia have compiled ‘significance thresholds’ (GFS) to assess groundwater contaminated with PFC. The GFS serve as criteria for the decision whether actions to remediate polluted groundwater are necessary. Thirteen of these PFC had been detected in groundwater at levels above their limit of quantitation and were assigned first priority.

**Results:**

The data regarding human health effects were sufficient to derive guide values according to the criteria of the German Drinking Water Ordinance for 7 of the 13 first-priority PFC. With regard to available ecotoxicological data, predicted no-effect concentration values from official risk assessments existed for 2 of the 13 first-priority PFC. A predicted no-effect concentration for protection of the aquatic biocenosis could be derived for eight more substances.

**Conclusions:**

After evaluation of data from available literature regarding both human health and ecotoxicological effects, significance thresholds ranging from 0.06 to 10.0 µg/L could be derived for 7 of the 13 priority PFC in groundwater. As a practical guide valid solely for human health-based values, a summation rule was proposed for exposures to mixtures of these seven PFC.

## Background

### Introduction

Per- and polyfluorinated chemicals (PFC)^1^ do not occur naturally in the animate and inanimate environment and are, therefore, of anthropogenic origin. Their chemical structure is generally characterized by a hydrophilic functional group and a hydrophobic fluorinated carbon chain giving them amphiphilic properties. Perfluorinated chemicals are organic molecules in which all hydrogen atoms have been substituted by fluorine atoms, with the exception of hydrogen atoms as constituents of functional groups. Polyfluorinated chemicals, in contrast, are organic molecules in which some, but not all hydrogens have been substituted by fluorine atoms. Perfluorinated chemicals are chemically very stable and metabolically either completely stable or barely biodegradable so that they can be classified as persistent substances. What is more, they can bioaccumulate and are subject to biomagnification. Certain polyfluorinated chemicals which can undergo physicochemical or biological processes that can transform them into perfluorinated chemicals with the physicochemical properties of this group of substances are referred to as precursor substances.

Because of their unique properties, PFC are employed in a multitude of uses. Certain compounds are used for manufacturing fluoropolymers such as PTFE (polytetrafluoroethylene) and side-chain fluorinated polymers. Those polymers as well as PFC mixtures are used, e.g. in the textile industry for the manufacture of water and dirt repellent, breathable fabrics, and in the paper industry for the production of soil-, fat- and water-repellent papers. PFC are used, e.g. in food packaging, for impregnating furniture, carpets and clothing, including shoes. Furthermore, PFC are used as anti-fogging sprays for glass, in antistatic materials and in semiconductors, as additives for cement, in cleaning agents, coatings, insulators, cosmetics, paints, household detergents, in fire-fighting foams, in pesticides, hydraulic fluids and non-stick surface coatings, e.g. for cookware.

Exact numbers on the production and circulation of individual compounds are generally unavailable, in particular because the content and/or use of these compounds in products or mixtures is not always labeled [4]. Therefore, importers are not always informed or aware of the possible presence of PFC in products.

As a result of their wide range of uses since the 1970ies and their persistence in the environment, PFC are detectable in water, soil, effluent sludge, biological waste, food, in aquatic and terrestrial life forms as well as in human matrices such as blood and breast milk. Due to the amphiphilic character of PFC, it can be assumed that the predominant path of distribution is via water. Effluents of industrial processes represent a further path of entry as they flow directly into communal waste water treatment plants (WWTP). In spite of complex filtration systems involving multiple stages, WWTP are unable to retain PFC. On the other hand, PFC may be mobilized by precipitation in soil, waste sites or in sewage sludge, which is used as “soil conditioner” in some areas of Germany, and thus enter into surface water [5].

According to the length of their perfluorinated carbon chain, these chemicals are grouped into long-chain (≥ 6 perfluorinated carbon atoms) and short-chain PFC (˂ 6 perfluorinated carbon atoms). Predominantly PFC with up to 10 carbon atoms are found in groundwater (Table 1).

**Table 1:** Findings of PFC in aqueous samples

Matrix	Number of measurements (n)	Substances^a^	Concentration range^b^ (ng/L)	Literature
Effluent/landfill leachate	24 24 24 24 24 24 24 24 24	PFBA PFPeA PFHxA PFHpA PFOA PFNA PFBS PFHxS PFOS	< LOQ—45,917 < LOQ—1781 < LOQ—4256 < LOQ—961 < LOQ—3260 < LOQ—211 < LOQ—17,686 < LOQ—537 < LOQ—1830	[88]
Surface water Ruhr area	22 22 22 22 22 22 22	PFBA PFPeA PFHxA PFHpA PFOA PFBS PFOS	< LOQ—143 < LOQ—1638 < LOQ—1248 < LOQ—148 < LOQ—3640 < LOQ—71.0 < LOQ—193	[89]
Surface water River Rhine and selected tributaries	38 38 38 38 38 38 38	PFBA PFPeA PFHxA PFHpA PFOA PFBS PFOS	< LOQ—3.0 < LOQ—42.0 < LOQ—77.0 < LOQ—11.0 < LOQ—48.0 < LOQ—46.0 < LOQ—152	[89]
Ground water	2057 2057 2057 2057 2057 2057 2057 2057 2057	PFBA PFPeA PFHxA PFHpA PFOA PFNA PFBS PFHxS PFOS	< LOQ—52.0 < LOQ—47.0 < LOQ—95.0 < LOQ—57.0 < LOQ—67.0 < LOQ—18.0 < LOQ—58.0 < LOQ—150 < LOQ—180	[90]
Drinking water	26 26 26 26 26 26 26 26 26	PFBA PFPeA PFHxA PFHpA PFOA PFNA PFBS PFHxS PFOS	< LOQ—4.4 < LOQ—5.2 < LOQ—6.4 < LOQ—1.5 < LOQ—6.1 < LOQ—1.4 < LOQ—5.8 < LOQ—12.1 < LOQ—4.7	[91]

*LOQ* limit of quantitation

^a^The substances listed were detected in amounts above the LOQ. Abbreviations cf. Tables 2 and 3

^b^These values represent the range between the lowest and highest detected amounts of the respective substances

Because PFC are found in waste water, surface water, groundwater, and drinking water (examples see Table 1), the German Environmental Agency [6–8] and the Federal Institute for Risk Assessment [9] assessed the human health effects of the two PFC indicator substances perfluorooctanoic acid (PFOA) and perfluorooctanesulfonic acid (PFOS), and issued a TDI^2^ of 0.1 µg/(kg day) and, together with the German Drinking Water Commission [10], a drinking water guide value of 0.3 µg/L for both and for the sum of their concentrations. These values were subsequently introduced by decree for the assessment of contaminated groundwater in several German states such as Baden-Wuerttemberg [11–13], Bavaria [14], and North-Rhine-Westphalia [15].

There are profound differences in biological half-lives between PFC of different chain lengths and in different species, in man, non-human primates and rodents. The elimination half-time of PFOA and PFOS is approximately 4 years in humans compared with days or hours in rodents. On the basis of these differences, Lud et al. [16] developed criteria for the assessment of the human health risk of short-chain PFC. These criteria were adopted and included in these early regulations.

As can be seen in Table 1, PFC above the LOQ were detected in all aqueous matrices (waste water, surface, ground- and drinking water). The highest concentrations were detected in unfiltered effluent, up to 45,917 ng/L (PFBA), surface water, up to 3640 ng/L (PFOA), groundwater, up to 180 ng/L (PFOS) and drinking water, up to 12.1 ng/L. In general, it can be said that the PFC concentrations decline in a sequence from effluent, surface water, groundwater to drinking water.

### Assignment

Since at this early stage ecotoxicological criteria for most of the PFC were missing and the literature evaluated concerning human health criteria included data only up to 2008, the German States’ Water and Soil Consortia (LAWA and LABO, respectively) decided in 2013 to derive updated criteria for the assessment of contaminated groundwater. A subcommittee appointed by these consortia compiled ‘significance thresholds’ (GFS) to assess groundwater contaminated with PFC. The PFC-subcommittee met eight times from November 2013 to February 2017 and evaluated publications and internet information collected by the German Environment Agency and the Environment Agencies of the States of North Rhine-Westphalia, Hesse, Bavaria, and Baden-Wuerttemberg, as well as the extensive literature collection of the Hessian State Laboratory (LHL).^3^ Further data resulted from the general exchange of experience of eight German Federal States and specific cases of contaminated sites, especially from North Rhine-Westphalia, Baden-Wuerttemberg, Bavaria, and Hesse.

The work order for the subcommittee comprised five sectors:

- Selection of relevant compounds (sections: “Chemical analysis” and “List of contaminants selected”).
- Compilation of the literature on human health and ecotoxicological effects (sections from: “Ecotoxicological data” up to “General description of human health effects and ambiguities”).
- Identification and possibly closing of data gaps.
- Derivation of *significance thresholds* for single compounds and, where appropriate, groups of compounds (sections from: “Summary of human health effects” up to “Outlook”).
- Preparation of a report including data sheets for 13 priority PFC, 7 of these with GFS values [17] (sections from: “Background” up to “Outlook”).

This paper describes problems encountered and solutions found for analyzing a group comprising thousands of PFC, the selection of relevant compounds, human health and ecotoxicological risk assessment resulting in the derivation of significance thresholds (GFS) for groundwater contaminated with PFC in Germany.

## Methods

### Chemical analysis

Because of the polar, hydrophilic functional group of PFC, analysis is performed by LC–MS/MS. In principle, PFC can also be derivatized and determined by GC–MS/MS, which, however, requires considerably greater effort in sample preparation. A standard method [18] has been developed in Germany for measurement of aqueous matrices and applies to ten perfluorinated compounds, for which validation characteristics were collected in a round robin test. This DIN-standard method has, in the meantime, been successfully applied in both private and state-run laboratories for routine measurements in aqueous matrices. Meanwhile an ISO-standardization project ISO/CD 21675^4^ has been instituted for detection of PFC in aqueous matrices encompassing analysis of 27 PFC. In addition to the PFC included in both DIN standards [18, 19], these 27 include further perfluorinated alkyl carboxylic acids and alkyl sulfonic acids, a telomer alcohol and telomer carboxylic acids and two polyfluoroalkyl phosphate esters.

The obvious expansion of additional substances within the framework of the ISO project cannot, from the point of view here, hide the fact that there are no analytical methods for a great portion of the approximately 3000 other PFC that have been released into the environment [20]. In fact, not even semi-quantitative methods exist for these substances, so that presently no one can estimate which PFC, in which concentrations exist in the environment and consequently in the food chain. In addition, the fact must be considered that quantification of PFC as a rule is, or must be, carried out using ^13^C-labeled internal standards to compensate for the influence of the particular matrix during sample preparation. Therefore, to assure the required validity of test results that will stand up in court it would be desirable to deploy an internal isotope standard for each and every analyte. The sheer number of analytes that come into question, the variety of necessary analytical LC–MS/MS methods, e.g. different polarities of the analytes, volatilities, functional groups as well as the elaborate quantification in view of ^13^C-labeled standards lead to the conclusion that this will not be possible with a justifiable effort and resultant acceptable cost.

The discussion about the determination of indicative or key parameters is an old one; which criteria should be chosen, however, is questionable. Conceivable would be, for example, selection according to functional group, the concentration that occurs in the environment, or the toxicity of each substance (which, however, with only few exceptions has not been assessed). In the meantime national and international research groups, as well as DIN, are working on the development of methods for the determination of cumulative parameters, e.g. similar to the AOX (Adsorbed Organic Halogen) method. In this sense, the EOF method (Extractable Organic Fluorine) aims at the cumulative determination of PFC in solid samples such as soil or sediments. The AOF method (Adsorbed Organic Fluorine), on the other hand, is used to detect organic fluorine compounds as cumulative parameters in aqueous samples. At present, it would appear that both EOF and AOF offer the possibility of establishing a cumulative parameter “total organic fluorine”. The question remains, however, how to evaluate the measurements obtained by these methods. For the environmental sector^5^ there are, with only one exception,^6^ no permissible limits or reference values for individual PFC or for cumulative parameters.

### List of contaminants selected

From among ca. 3000 possible PFC contaminants [20] 23 environmentally relevant substances were selected. Selection criteria for this group were both PFC presence in the environment and available analytical techniques. Thirteen of these had been detected in groundwater at levels above their LOQ and were assigned first priority (Table 2). Apart from PFHpS, H_4_PFOS, and PFOSA, all these substances are listed in the German analytical standard procedure DIN 38407-42 [18] and their method specifications have been determined in a round robin test.

**Table 2:** First priority PFC, listed in DIN 38407-42 [18]; except PFHpS, H_4_PFOS, PFOSA

No.	CAS-no.	Name	Abbreviation
1	375-22-4	Perfluorobutanoic acid	PFBA
2	2706-90-3	Perfluoropentanoic acid	PFPeA, PFPA
3	307-24-4	Perfluorohexanoic acid	PFHxA
4	375-85-9	Perfluoroheptanoic acid	PFHpA
5	335-67-1	Perfluorooctanoic acid	PFOA
6	375-95-1	Perfluorononanoic acid	PFNA
7	335-76-2	Perfluorodecanoic acid	PFDA
8	375-73-5	Perfluorobutanesulfonic acid	PFBS
9	355-46-4	Perfluorohexanesulfonic ac	PFHxS
10	375-92-8	Perfluoroheptanesulfonic acid	PFHpS
11	1763-23-1	Perfluorooctanesulfonic acid	PFOS
12	27619-97-2; 425670-75-3 (Anion)	1*H*,1*H*,2*H*,2*H*-Polyfluorooctanesulfonic acid, H_4_-Polyfluorooctanesulfonic acid, 6:2 Fluorotelomer sulfonic acid, FTS	H_4_PFOS, 6:2 FTSA
13	754-91-6	Perfluorooctanesulfonamide	PFOSA or FOSA

The remaining ten longer chain PFC were grouped as second priority (Table 3). They can be analyzed and accumulate in biota, but did not exceed the LOQ in groundwater samples. For these substances the derivation of GFS was not planned for the present. The priorities are to be updated according to the progress of the analytical techniques.

**Table 3:** Second priority PFC

No.	CAS-no.	Name	Abbreviation
14	2058-94-8	Perfluoroundecanoic acid	PFUnA, PFUdA, PFUndA+D53
15	307-55-1	Perfluorododecanoic acid	PFDoA
16	72629-94-8	Perfluorotridecanoic acid	PFTrA, PFTrdA
17	376-06-7	Perfluorotetradecanoic acid	PFTeA, PFTetA
18	335-77-3	Perfluorodecanesulfonic acid	PFDS, PFDeS
19	34598-33-9	2*H*,2*H*,3*H*,3*H*-Polyfluoroundecanoic acid	H4PFUnA
20	1546-95-8	7*H*-Dodecanefluoroheptanoic acid	HPFHpA
21	34598-33-9	2*H*,2*H*-Polyfluorodecanoic acid	8:2 FTA, 8:2 FTCA, n-8:2FTCA, H2PFDA
22	757124-72-4; 414911-30-1 (Anion)	1*H*,1*H*,2*H*,2*H*-polyfluorohexanesulfonic acid	4:2 FTSA, H4PFHxS
23	39108-34-4; 481071-78-7 (Anion)	1*H*,1*H*,2*H*,2*H*-polyfluorodecanesulfonic acid 8:2-fluorotelomer sulfonic acid	8:2 FTSA, H4PFDS

The CAS^7^ numbers listed, strictly speaking, designate only the non-branched, linear compounds. De facto, the technical mixtures contain only 70–80% of the non-branched plus 20–30% of various branched compounds. These differ in their physicochemical properties as well as their bioavailability and their degradability in the environment [22]. A further distinction, however, is impossible given the multitude of theoretically possible and practically occurring single compounds. Tables 2 and 3 contain the undissociated carboxylic and sulfonic acids that had been analyzed at the time of prioritization (2013). In the environment, these compounds mostly exist as anions, because they are either strong acids themselves (as the short-chain PFC) or they react with strong bases. Therefore, the results of the (eco)toxicological tests mostly refer to the anions.

### General Conditions for Risk Assessment of PFC

In the EU, some PFC have been identified as substances of very high concern (SVHC) according to Article 57 of the REACH regulation (EC) No 1907/2006 [23] by unanimous agreement of the Member State Committee. Perfluorinated carboxylic acids containing 8–14 carbons in a chain were added to the REACH-Candidate List: PFOA, PFNA, and PFDA are persistent (P), bioaccumulative (B), and toxic (T), and are, therefore, designated as PBT substances under REACH. The longer chain perfluorocarboxylic acids PFUnA, PFDoA, PFTrA, and PFTeA are considered to be very persistent and very bioaccumulative substances, so-called vPvB substances. The properties that lead to an SVHC identification are listed in Annex XIII of the REACH regulation.

Because of the poor degradability of PBT and vPvB substances, there is concern that effects in organisms cannot be predicted in the long run. Accumulation in organisms is virtually irreversible and the content of these substances in the environmental compartments can only very slowly be reduced by lowering the emissions. Moreover, some PBT and vPvB substances, such as PFC, have the potential to pollute remote areas through long-range transport via air and water. Even when standardized tests in the laboratory show no toxic effects, chronic effects may occur through long-term exposure to low doses. Because of the long life cycles of organisms at the end of the food chain, these effects will hardly be predictable. And the concentrations of PBT and vPvB substances in man and the environment cannot be predicted over long periods of time. This is another reason why chronic effects cannot be excluded. Therefore, according to the principles of REACH, for PBT and vPvB substances no safe levels can be derived for the environment [24].

### Assessment criteria specific for contaminated groundwater

The concept of “detrimental change” of a body of groundwater in the German Water Act [25] is substantiated for a single contaminant by the “significance threshold” (GFS). The GFS serve as criteria for the decision whether actions to protect the quality of groundwater or to remediate polluted groundwater are necessary [26]. A detrimental alteration of the water is defined as “changes of the properties of a body of water, which impair the public welfare, especially the public water supply” (§ 3). The purpose of the law is “to protect a body of water against detrimental changes of its properties… and to compensate (as much as possible) impairments, that are not insignificant (§ 6). All activities are to be carried out in such a way that “a detrimental change of the water quality should not be feared.” Examples are “groundwater pollution abatement” (§ 48) and the “handling of substances hazardous to water” (§ 62). Therefore, a groundwater contamination can be classified as insignificant [27] if it is not ecotoxic (criterion 1: intact habitat) and fulfills the requirements (including the limit values) of the German Drinking Water Ordinance (criterion 2: [28]) or values derived accordingly [criterion 3: harmless to human health (§ 6, 1; [28]) and esthetically unobjectionable (§ 4, 1; [28]).

**Fig. 1:**
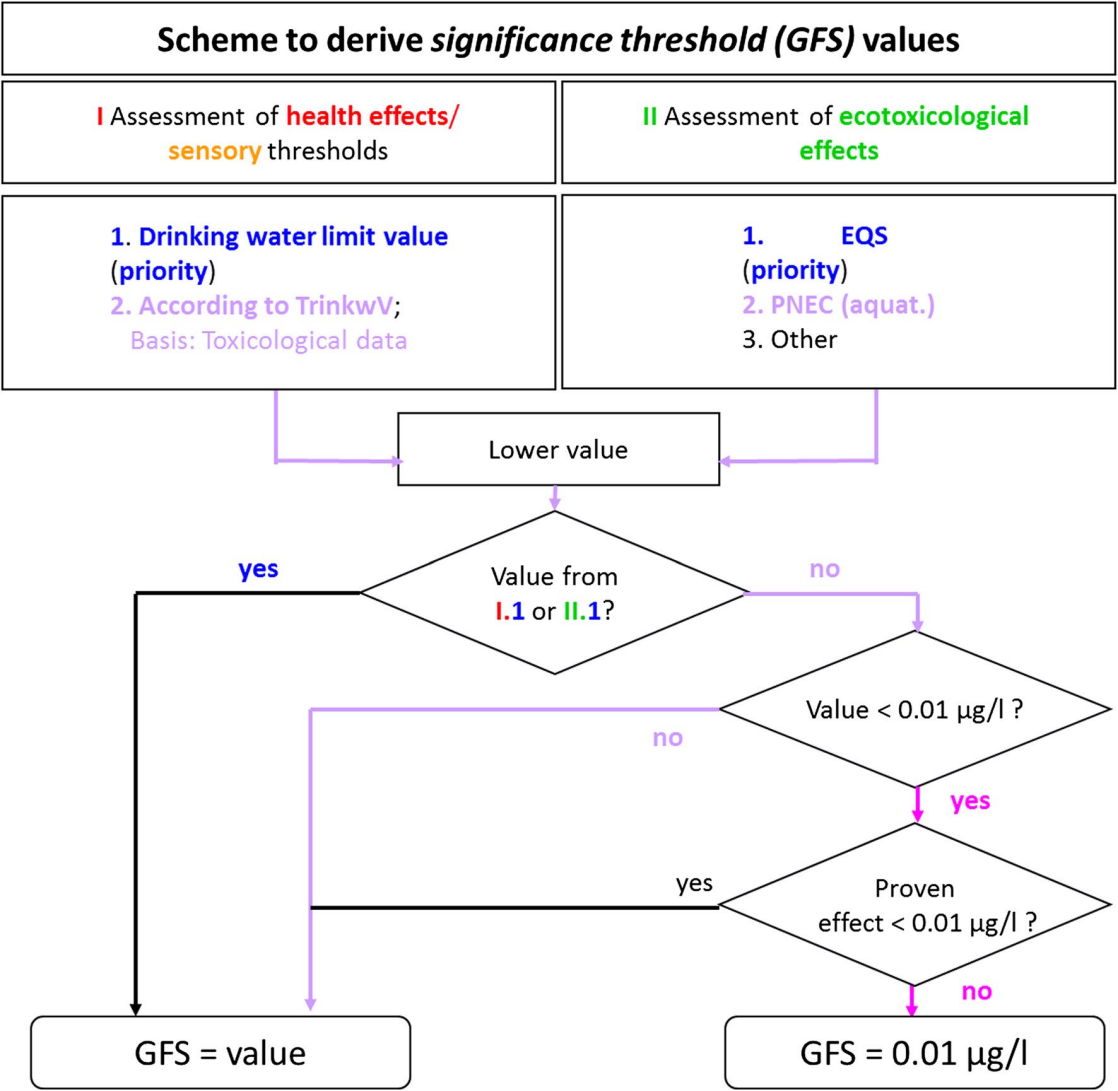
Scheme to derive significance threshold (GFS) values [62]

The assessment according to the German Drinking Water Ordinance and the ecotoxicological assessment are carried out in parallel (Fig. 1). In both cases the highest dose or concentration without measurable effect (NOAEL)^8^ has to be determined. The lower value of the two origins (German Drinking Water Ordinance or ecotoxicology) is relevant. In both cases effective laws [limit values or environmental quality standards (EQS)] have the highest priority (1). Values based on data from the toxicological literature have a lower priority (2). If the lower value is taken from an effective law, it becomes the significance threshold without further consideration.

Toxicological data from animal experiments are extrapolated to the human equivalent dose which can then be converted into a drinking water concentration (section: “The standard scenario”). Second priority values are checked whether these are lower than 0.01 µg/L. This value serves as the lower limit of the GFS. Significance thresholds lower than this value are possible only if they are based on a proven effect at this concentration (not an assessment factor). The lower limit was introduced because the ecotoxicological evaluation often leads to lower values than the human health-based values, on one hand, and values below the LOQ are not meaningful, on the other hand [26].

PNEC^9^ values are calculated by dividing the lowest NOEC^10^ of the most sensitive species (algae, crustaceans, fish) by an assessment factor of 10 or greater, depending on completeness of the data set [29]. Values derived according to the German Drinking Water Ordinance are based on the TDI (for non-carcinogens) or an added lifetime risk of 10^−6^ (for carcinogens) or the odor detection threshold, whichever is the lowest value.

### Scenarios for human exposure related to groundwater

The assessment of the risk from a toxic chemical substance entails the combination of the effect level (which indicates the chemical’s hazard potential) with data describing the exposure of an organism. PFC can reach a human target organism via several pathways of exposure (food, air, drinking water). With respect to groundwater, of course, the intake of drinking water is a major route of human exposure.

#### The standard scenario

As a rule only 10% of the toxicologically acceptable total dose is allocated to drinking water as exposure pathway [30]. The remaining 90% is allotted to other possible pathways, such as uptake via food. The contribution of drinking water may, however, be increased if scientific data suggest a greater portion of the total intake via this pathway. The standard assumptions for converting the acceptable or tolerable dose (ADI^11^ or TDI^2^) into a concentration in drinking water (LW^12^) for adult humans are a daily uptake of 2 L of water and a body weight of 70 kg.

#### Unaccounted-for scenarios

The irrigation of *crops* with groundwater or surface water is an essential element of food production in Germany and other European countries. In this context, especially short-chain PFC present in the irrigation water may accumulate in plants. This may lead to an increased exposure of humans via the food chain, possibly in excess of tolerable daily doses [31].

The accumulation of certain PFC in fish is documented in numerous studies [32–36]. Thus, the exposure of humans is possible through the consumption of contaminated fish whenever *fish-rearing ponds* are fed by groundwater or spring water contaminated with PFC [37].

For *surface waters,* a limit value of 0.65 ng PFOS/L ([17, 38]—details in the PFOS data sheet page 65) entered into force on September 13, 2013 as EQS for the protection of human health with regard to the consumption of fisheries products within the framework of the EQS directive [39]. The European Scientific Committee on Health and Environmental Risks has confirmed the fisheries-derived EQS as the lowest limit for PFOS [40]. This fisheries-based EQS is not relevant for groundwater, because according to the legal procedure [17] the human exposure via the food chain, via crop irrigation, and via the accumulation in fish from contaminated groundwater/spring water is not considered. The human health-driven objective of the significance thresholds is that “everywhere the groundwater shall remain usable for human consumption as drinking water”. The protection of human health during the consumption of fish is not considered in deriving the threshold values, because fish consumption has no bearing on the groundwater.

The exposure scenarios via the use of groundwater contaminated with PFC for the irrigation of crops or via the consumption of fish from waters whose influx from groundwater contains measurable concentrations of PFC, therefore, need a special human health-based assessment. And even with groundwater complying with the GFS value for PFOS (100 ng/L), care has to be taken that the EQS of 0.65 ng/L in the surface water is not exceeded by seepage of contaminated groundwater into a watercourse.

### Ecotoxicological data

#### Search for data

For the ecotoxicological evaluation, effect data from the individual trophic levels were recorded after systematic research in the following sources:

- Databanks with ecotoxicological entries: ECOTOX (US EPA), ETOX (German Environmental Agency-UBA), HSDB.
- REACH registration dossiers of the European Chemicals Agency (ECHA).
- Literature collection of the LHL (cf. section: “Assignment”).
- Special reports and reviews.
- Recent experimental results of the Bavarian State Agency for the Environment [14] from a project to evaluate contaminants specific for a river catchment in the context of the water framework directive.

#### Exclusion of invalid data

For deriving a PNEC (see footnote 9) minimum quality criteria have to be met according to European guidelines [24, 29]. These are evaluated according to Klimisch et al. [41]. Studies or data from the literature, for which only insufficient experimental details are known, and which are only listed in secondary literature or in abstracts (validity 4: reliability indeterminable) are not usable, as a rule, to derive an authoritative GFS value. The same is true for insufficiently documented studies, which do not stand up to expert scrutiny, for instance because the instructions of a test method were not followed (validity 3: not reliable).

As far as appropriate and possible, the data searched for were examined for their reliability and validity. In case of obvious violations against the instructions of the test applied, or in case of justified doubts in the correct performance of a study, the study results were not included in the PNEC derived. Especially, the short-chain perfluorinated alkylsulfonates are strong acids which can cause pH values down to < 2 at the concentrations used in the tests. All established operation procedures include a pH adaptation where required, e.g. by addition of NaOH, to eliminate mortality or adverse effects solely due to the acidity. All publications of ecotoxicological tests with the PFC considered here were checked in this respect. Some publications contain no statement whether such pH adjustment was performed, or about the prevailing pH value during the test. The corresponding test results are, therefore, not meaningful and were not considered. In some cases relevant information could be obtained from authors; in case of compliance with pH restrictions the results reported in such publications were regarded as valid.

## Results—outline of effect data used to derive GFS values

### Human health evaluation

#### Use of cell test data

Some PFC displayed endocrine effects in laboratory tests with mammalian cells. Experiments with fish gave similar results. Rosenmai et al. [42] showed that short-chain PFC did not exert such effects, as opposed to the long-chain representatives. Studies in this regard are reported in the data sheets [17, 38]; the results, however, cannot be quantified in terms of a NOAEL (see footnote 8) Therefore, they have not yet found their way into the derivation of human health-based GFS values.

Through the observation of malformations or other serious effects on embryos of aquatic animals, substances acting in this way can be identified at an early stage in screening tests aiming at the reduction of tests with mammalian organisms [43, 44] such as the zebrafish egg test [45] or the chronic test with clawed frogs ([46]; No. 231). Such results cannot be used either for the human health-based GFS values, because they lack a NOAEL.

#### Main source

Because of the seriousness of the problems caused by PFC, a high and steadily growing number of publications deal with the implications of this class of compounds. Assessments and reviews carried out earlier facilitate the survey of the toxicological profile of these substances. The US Agency for Toxic Substances and Disease Registry has produced an elaborate and up-to-date characterization of PFC (especially PFOA and PFOS) with respect to their toxicity [3]. Information from the respective report is included in the following sections.

#### General description of human health effects and ambiguities

##### a. Animal data

Many of the adverse health effects of PFC observed in experimental animals are attributed to the ability of the PFC to activate the peroxisome proliferator-activated receptor α (PPARα). PPARα regulates several physiological processes, among others fatty acid oxidation in liver; it is also presumed to exert an influence on reproduction and child development [47], and is thought to be associated with the induction of tumors in the liver of rodents by non-genotoxic carcinogens [48].

Species differences were found in the reaction to PPARα agonists: rats and mice are the most sensitive species, whereas rabbits, non-human primates and humans are less sensitive. These differences in sensitivity might be explained by differences in the inducibility of PPARα by exposure to a peroxisome proliferator or differences in the tissue-specific expression of PPARα.

Activation of the receptor in rodents is followed, mainly—but not exclusively—in liver, by a sequence of biochemical and morphological events. These include a hepatocellular hypertrophy through an increase in number and size of peroxisomes, a strong increase of the peroxisomal β-oxidation of fatty acids, an increased CYP450-mediated ω-hydroxylation of lauric acid as well as alterations in lipid metabolism.

The weight of the liver and the parameters of fatty acid β-oxidation were generally increased with increasing PFC chain length up to about ten carbon atoms. Significant peroxisome activity seems to require a carbon chain length of greater than seven, but a *slight rise* above the control level was reported with a chain length of *four carbon* atoms already. It also appeared that the differences in activity are not directly related to the carbon chain length as such but rather to differential accumulation in the liver. As with PFOA and PFOS, also with PFDA effects regarding an increased fetal mortality were observed [49]. In contrast, gestational exposure to the shorter chain PFBA or PFHxS had no effect on the survival or the body weight of the offspring. Decreases in spontaneous activity followed by an increase in activity were observed in mice exposed to PFHxS up to postnatal day 10; no alterations were observed in mice similarly exposed to PFDA.

Studies of the immunotoxicity of PFC in rodents indicate a considerably higher sensitivity of mice compared with rats. The immunological changes induced in adult mice by PFOA and PFOS appeared as atrophy of thymus and spleen, alterations in thymocyte and splenocyte phenotypes, and impaired response to T-dependent antigens.

##### b. Comparison of animal and human sensitivity

Studies with PPARα-null-mice suggest, however, PPARα-independent mechanisms of PFOA and PFOS toxicity as well. Thus PFOA induced hepatomegaly to the same extent in wild-type mice and PPARα-null mice, but failed to increase acyl-CoA oxidase activity in PPARα-null mice. Also the PFOA-exposure of monkeys resulted in an increased absolute liver weight, which was partially associated with a significant mitochondrial, but not with peroxisomal proliferation.

Some developmental effects observed in animals have not been observed in humans. A gestational exposure study of PFOA conducted using wild-type, PPARα-null, and PPARα-humanized (expressing human PPARα) mice showed that postnatal survival was lower in wild-type, but not in null or humanized mice [50].

In cultured rat, mouse, and human hepatocytes, perfluoroalkyl sulfonate compounds were less potent than perfluoroalkyl carboxylate compounds in stimulating the PPARα-induced gene expression, and the potency of stimulation increases with carbon chain length [51, 52]. PFOS (or PFOA) significantly increased activation of mouse PPARα/β/δ (PFOA also of human PPARα) relative to vehicle control, but not the human PPARβ/δ. PFOA, like many other PPARα-agonists, induced hepatocellular adenomas, Leydig cell adenomas, and pancreatic acinar cell adenomas in rats.

An extensive review of the literature concluded that although humans possess sufficient PPARα to mediate the human hypolipidic response to therapeutic fibrate drugs, there are enough differences (in gene promoters, receptors, activities, and receptor levels) between the response of the human liver to PPARα agonists and that of rats, so that for liver tumors in animals and in man most likely a different mechanism has to be assumed [3]. Because of insufficient data, however, this conclusion remains uncertain. Generally speaking, humans seem to react less sensitively to the health effects of the first-priority PFC than laboratory animals, especially mice.

##### c. Epidemiological studies of human populations

Epidemiological studies of human populations show statistically significant associations between serum PFC levels (especially for PFOA and PFOS) and a multitude of concentration-dependent health effects, even if these were not always consistent across studies. The association of the PFOA and PFOS serum level was consistent with elevated lipid concentration in the serum, raised uric acid levels, reduced birth weight, and altered biomarkers for liver damage. There is also equivocal evidence for carcinogenicity.

Concerning the biomarkers for possible effects on the liver, no consistent associations between serum liver enzymes (primarily alanine-aminotransferase—ALT, aspartate-aminotransferase—AST, and γ-glutamate transferase—GGT) and the PFOA or PFOS concentration in the serum appeared in workplace studies. A study with highly exposed subjects of the general population found significant connections between PFOA and PFOS in serum and the ALT and bilirubin-concentrations, while the extent of the effect was rated as probably not biologically relevant.

Studies with rats, mice and monkeys have identified the liver as one of the toxicologically most sensitive target organs. The data with humans, however, are not so significant, while the PFOA and PFOS levels in serum were much lower than those that gave rise to effects in experimental animals.

The main weakness of the epidemiological studies with human subjects stems from the fact that PFOA and PFOS, the indicator PFC, have meanwhile been globally distributed and are ubiquitously present in the environment. The effects of a single PFC can, therefore, not be studied without serious interference from other PFC which are simultaneously present [17, 53, 54]. ATSDR [3] conclude: “*Studies with highly exposed residents and the general population have often reported significant associations for both PFOA and PFOS, and the possible interaction of the various PFC with the health endpoint of concern is not known. …the mechanisms of toxicity of the observed health effects have not been established and these effects have not been reported in laboratory animals. Serum cholesterol and other lipid levels are also affected by PFOA and PFOS exposure in rats and mice; however in rodents, exposure to PFC resulted in significant* ***decreases*** *in serum lipid levels* (as opposed to **elevated** lipid levels in humans). *These uncertainties preclude the use of currently available epidemiology studies as the basis for developing an MLR* (= quantitative assessment criteria) *for PFOA or PFOS*.”

#### Summary of human health effects

The available data were sufficient to derive guide values according to the criteria of the German Drinking Water Ordinance for 7 of the 13 PFC of first priority. Table 4 gives an overview of pivotal studies, and effects relevant for deriving the human health-based drinking water concentrations as a base for these seven GFS values derived. For each substance the lowest NOAEL is listed as point of departure. Using the ratio of elimination half-life human/animal, and further assessment factors, it is extrapolated to a human equivalent dose which is then converted into a concentration in drinking water. The last column shows the effect of rounding. For more detailed information, the reader is referred to the original reports by LAWA [17] and UBA [38].

**Table 4:** Overview of the rationales for PFC with GFS

No.	Abbreviation	Pivotal study	Effect at LOAEL	Point of departure (mg/kg day)	Factor elimination t_1/2_ human/animal	Assessment factors 1. Duration 2. Inter-dyn. 3. Intra	Human equivalent dose (μg/kg day)	Concentration in DW (µg/L)	GFS (μg/L)
1	PFBA	90 days oral, rat ♂ [92, 93]	Hepatocellular hypertrophy; thyroid hyperplasia/hypertrophy	6	8	10 2.5–1 10 –––– 250–100	3–7.5	10–52^a^	10
3	PFHxA	104 weeks oral, rat ♂ ♀ [77, 107–109]	Lower urine pH, kidney histology	15	327	– 2.5 10 –––– 25	1.84	6.42	6
5	PFOA	Human [94] supported by 26 weeks oral, monkey ♂ [95]; 17 days oral mouse ♀ [53]	Reduced protection against A/H3N2-virus provided by influenza vaccination [94]. Supported by elevated liver weight [95]; delayed mammary gland development [53]	Serum concentration at steady state: 90 µg/L human serum, converted to human equivalent dose			0.02037	0.071	0.1^c^
6	PFNA	2 generation oral, rat ♂ ♀ [96, 97]	Microscopic liver lesions; hepatocellular hypertrophy	0.025	50	3* – – – 10** –––– 30	0.0167	0.0583	0.06
8	PFBS	90 days oral, rat ♂ [98]	Reduced erythrocytes, hemoglobin and hematocrit	60	146	10 2.5 10 –––– 250	1.64	5.74	6
9	PFHxS	42 days oral, rat ♂ [99]	Raised liver weight, hepatocellular hypertrophy; thyroid hyperplasia; reduced hematocrit	1	90	15 2.5 10 –––– 375	0.03	0.1037	0.1^b^
11	PFOS	2 years oral rat ♂ [100]; 26 weeks oral monkey ♂ [3, 101]; 420 days oral monkey [102]; 28 days oral mouse ♂ [103–106]	Hepatocellular hypertrophy [100]; low cholesterol [3, 101]; low cholesterol [102]; increased NK activity, inhibited IgM production [103–106]	Serum concentration at steady state: 20 µg/L human serum as middle from the pivotal studies [17, 38], converted to human equivalent dose			0.0286	0.1	0.1

Assessment factors for: 1. duration of the study, 2. inter-species toxico-dynamic differences, 3. intra-species differences, * extrapolation to NOAEL, ** special risks such as carcinogenicity, genotoxicity, etc. Under the line: product of factors

*NK* natural killer cells, *IgM* immunoglobulin M

^a^Drinking water allocation 10–20%

^b^Secured by identical HRIV

^c^Value rounded because of mixed exposure with PFOS under field conditions

The evaluation of PFHxS, however, must be regarded as a borderline case. It is based not on a 90-day study as required, but on a study with 42 days of exposure only. The factor for lifetime extrapolation was, therefore, raised to 15. A consideration conducted in parallel aiming at a precautionary health-related indication value (HRIV)^13^ arrives at the same result of 0.1 µg/L for PFHxS. Therefore, this result can be accepted as a borderline case, especially regarding the need for a guide value.

The lowest value of 60 ng/L was calculated for PFNA. This value, though, contains a special safety factor of 10 because of the potential for toxicity for reproduction (classification Repr. 1B) and presumably carcinogenicity (classification Carc. 2). The highest value calculated so far was 10 µg/L for PFBA. It is only slightly higher than the value hitherto specified by the German Environment Agency (7 µg/L; [8]). Moreover, the values for the individual compounds seem to relate reasonably to one another.

### Ecotoxicological evaluation

#### Deriving PNEC values to protect the aquatic biocenosis

Except for PFOS and PFOA, for which official PNEC values are available from the European or international assessment of chemicals, PNEC values for the other PFC of first priority were derived according to the rules of the TGD [29]. In every case, the “assessment factor method” ([29]; section 3.3.1.1) had to be applied, since no sufficient effect data for the application of the statistical “species sensitivity distribution” method were available [17]. A decision whether a test result could be evaluated as long-term result was based upon the classification for the OECD Guidelines for Testing of Chemicals ([29]; Appendix 1, A1.3.2.10).

In some cases which could not be assigned to an OECD guideline, because the test had been performed according to a comparable method of a different organization, e.g. the US EPA, the result was regarded as chronic if the duration could be regarded as equivalent to a corresponding OECD guideline [17].

With regard to tests with effects on fish, several test guidelines for short-term tests have been issued in recent years, not least for animal welfare reasons. These include the fish short-term reproduction assay ([55]; Guideline 229), the fish sexual development test ([56]; Guideline 234), the fish embryo acute toxicity (FET) test ([57]; Guideline 236), and above all the so-called fish egg test according to DIN EN ISO 15088-T6 [45], all of which could not be considered in the TGD [29]. These tests can detect effects on early developmental stages of fish which hitherto could only be determined in chronic tests. But the results, as far as available in isolated cases, were only used as complementary information for deriving PNEC values. Especially with surface-active substances such as PFC, concerns may be raised that specific damage for adult fish such as an impairment of the gill function through lowering of the water’s surface tension cannot be adequately determined in early life stages. This can be seen in practice, e.g. with PFPeA which showed no effect in the fish egg test at as high a level as 4000 mg/L [58], while the acute toxicity for adult fish according to OECD 203 resulted in an LC_50_ of 32 mg/L [59], a value that is more than 100-fold lower.

#### Summary of ecotoxic effects

For 2 of the 13 PFC of first-priority PNEC values from official risk assessments were existent: 570 µg/L for PFOA [60] and 0.23 µg/L for PFOS [61]. A PNEC to protect the aquatic biocenosis could be derived according to TGD [29] for eight more substances. For two of these, the chronic toxicity data for three trophic levels are complete, so that for the derivation of the PNEC starting with the lowest effect value the minimal required safety factor of ten could be applied. For none of the remaining substances a higher safety factor than 100 was necessary. No PNEC could be derived for PFHpA, PFHpS, and PFOSA, because of lack of data either for one trophic level (fishes) or complete lack of data in the case of PFOSA. In summary, with increasing chain length a decreasing trend can be observed for the PNEC values of the perfluorinated sulfonic acids as well as for the perfluorinated carboxylic acids with one exception (PFPeA). As would be expected the toxicity increases with chain length [17].

### Human health values and ecotoxicological values compared

The aquatic biocenosis turned out to be less sensitive than the legal requirements of the German Drinking Water Ordinance in the case of those PFC for which GFS values could be derived [17]. In contrast, previous experience with GFS values shows that ecotoxic effects often occur at lower concentrations than effects on human health [26, 27, 62]. The higher toxicity of PFC for humans compared with aquatic organisms can be explained by their far longer elimination half-lives from human tissues.

### GFS values recommended

#### Individual substances

##### a. Substances sufficiently characterized

The GFS values listed in Table 5 are proposed for the PFC of first priority. The toxicological data for seven of the 13 first-priority PFC allowed the derivation of GFS values. The rationales elaborated by the LAWA/LABO-subcommittee [17] for the single substances should be kept in mind in making use of the values.

**Table 5:** GFS for PFC [17, 38] of first priority (rationale for prioritization see section “List of contaminants selected”)

No.	Name, abbreviation (CAS no.)	GFS (μg/L)	Basis (μg/L)	
			Human health	PNEC
1	Perfluorobutyric acid, PFBA (375-22-4)	10	10	1260
2	Perfluoropentanoic acid, PFPeA (2706-90-3)	–	− (HRIV: 3.0)	320
3	Perfluorohexanoic acid, PFHxA (307-24-4)	6	6	1000
4	Perfluoroheptanoic acid, PFHpA (375-85-9)	–	− (HRIV: 0.3)	–
5	Perfluoroctanoic acid, PFOA (335-67-1)	0.1	0.1	570
6	Perfluorononanoic acid, PFNA (375-95-1)	0.06	0.06	8
7	Perfluorodecanoic acid, PFDA (335-76-2)	–	− (HRIV: 0.1)	10
8	Perfluorobutanesulfonic acid, PFBS (375-73-5)	6	6	3700
9	Perfluorohexanesulfonic acid, PFHxS (355-46-4)	0.1	0.1	250
10	Perfluoroheptanesulfonic acid, PFHpS (375-92-8)	–	− (HRIV: 0.3)	–
11	Perfluorooctanesulfonic acid, PFOS (1763-23-1)	0.1	0.1	0.23
12	H4-Polyfluorooctanesulfonic acid, H4PFOS (27619-97-2)	–	− (HRIV: 0.1)	870
13	Perfluorooctanesulfonamide, PFOSA (754-91-6)	–	− (HRIV: 0.1)	–

The HRIV for non-assessable substances is explained in sections “Substances insufficiently characterized” and “Uncertainty analysis” as well as in LAWA [62]

##### b. Substances insufficiently characterized

Substances with insufficient data for a human health assessment were alternatively evaluated making use of the concept of the HRIV (see footnote 13) (*GOW*, [section 4.4 in 62]), which was developed by the German Environment Agency [1, 2] to safeguard the resource “drinking water” against contaminants, which cannot or can only partially be assessed concerning its harmfulness for human health (non-assessable substances). The HRIV can be regarded as the upper bound of the precautionary zone and the lower bound of the zone of concern. It is as high as possible and as low as necessary to compensate the lack of data. The German Environment Agency has created a criterion in the HRIV that includes precautionary aspects and experience gained from earlier assessments of drinking water contaminants to bridge the toxicological data gap.

The basic HRIV for non-assessable pollutants is 0.1 µg/L. With more exonerating data about a compound, its HRIV can be raised in a stepwise fashion up to 10 µg/L. For strong genotoxins the HRIV has to be lowered to 0.01 μg/L. As upper bound of the precautionary zone, the HRIV more than covers the human health-related aspects of the GFS, and a PNEC at or below the HRIV is sufficiently protective for groundwater as drinking water resource.

For the PFC considered here, the potency concerning human health was consistently greater than their ecotoxicological potency. Therefore, none of the PNEC values could be recommended as GFS, since all of them were clearly higher than the HRIV [17].

#### GFS vs. human biomonitoring values

The Human Biomonitoring Commission of the German Environment Agency has established HBM-I-values of 2 ng PFOA/mL blood plasma and 5 ng PFOS/mL blood plasma [63–66]. Human biomonitoring accounts for the total exposure via all possible routes and is, therefore, more realistic than the consideration of only one single route (e.g. drinking water). Human biomonitoring is, therefore, the preferable instrument for the assessment of total human exposure. Decisions about actions, however, where necessary at a later stage require the attribution of exposure to single routes. If human biomonitoring is possible, of course, the HBM-I-value applies as standard of comparison. The German Environment Agency after hearing the Drinking Water Commission of the Federal Ministry for Health in its “Update of the Provisional Assessment of Per- and Polyfluorinated Chemicals (PFC) in Drinking Water” [38, 67] endorses the assessments and conclusions presented here.

#### Grouping and summation

Prerequisite for an appropriate aggregation of PFC in groups is an identical mechanism of action. For this purpose little is known about PFC. Some hints are given in the reviews by Stahl et al. [32], Borg and Håkansson [68], the Danish EPA [69], and the ATSDR [3]. According to these, the ability of the PFC to activate the PPARα seems to be especially significant. As described in “General description of human health effects and ambiguities” section PPARα regulates the fatty acid oxidation in liver, exerts an influence on reproduction and child development, and is thought to be associated with the induction of tumors in the liver of rodents by non-genotoxic carcinogens. Because of their structural analogy to endogenous fatty acids, PFC are transported via a reversible bond to albumin in blood. They are also ligands of the PPARα of the cell nucleus. The binding to PPARα leads to peroxisomal proliferation and subsequently to the catabolism of fatty acids, especially in liver as the main organ for the storage and mobilization of lipids. Such peroxisome proliferators are the cause of hepatocellular hypertrophy and an increased liver weight.

Studies of the toxicity for reproduction and child development have shown, however, that neonatal mortality of mice after exposure in utero against PFOA and PFNA depended on PPARα, but exposure against PFOS was PPARα independent. For this endpoint, a mechanism unrelated to PPARα seems to be at least participating. Moreover, activation of nuclear receptors other than PPARα through PFC has been demonstrated, such as the constitutive androstane receptor (CAR) and the pregnane-X-receptor (PXR). Liver toxicity and the strength of induction of peroxisome proliferation in rats (where rodents react more sensitively to peroxisome proliferators than humans) furthermore depend on the carbon chain length. For a similar increase of the activity of hepatic acyl-CoA-oxidase as measure of proliferation 50 times higher doses of PFBS were necessary than of PFOA or PFHxS.

Until today no scientifically conclusive method for the assessment of human health effects of multiple substance exposures exists. Practical guidance relating to human health is given by the TRGS 402 for work place exposures [70]. TRGS 402 stipulates for the presence of multiple substances the generation of substance indices (*I*) from the measured values and the standards of evaluation such as

$$I = \frac{C}{\text{BM}}$$

(with *C* = measured concentration and BM = standard of evaluation), and the summation of these, in the present case with the human health-based GFS_h_ as BM, to an assessment index BI:

$${\text{BI}}_{{{\text{GFS}}_{\text{h}} }} = \sum {I_{i} } = \frac{{C_{1} }}{{{\text{GFS}}_{\text{h1}} }} + \frac{{C_{2} }}{{{\text{GFS}}_{\text{h2}} }} + \frac{{C_{3} }}{{{\text{GFS}}_{\text{h3}} }} + \ldots$$

If the assessment index BI > 1, the assessment standard for the sum is considered to be exceeded. Only concentrations greater than or equal to the limit of quantitation (≥ LOQ) are to be considered in this context. This pragmatic approach applies for human health as a legally protected good and for GFS values only so far as they are based on human health. Because of their special character (sections: “Substances insufficiently characterized” and “Uncertainty analysis”), HRIV are not included in the summation.

#### Uncertainty analysis

Of the seven PFC, for which GFS values along the lines of the German Drinking Water Ordinance [28] could be derived, only for one substance was the key study based on the minimum data set of a (subchronic) 90-day study. The other studies had been conducted for at least twice the exposure duration, twice for 2 years, and two 2-generation studies were available. Insofar the assessments can be regarded as sufficiently safe. The database for PFHxS has to be considered as critical because its exposure duration of 42 days was lower than the required 90 days for a subchronic test. But because the HRIV consideration supports the result, an underestimation of risk seems unlikely.

Some other assessment procedures provide special safety factors because of a lack of data, e.g. for developmental toxicology or immunotoxicology. Such factors are not conventional in the assessment procedure analogous to the German Drinking Water Ordinance, which may lead to an underestimation of the risk. With respect to the PPARα-agonistic effect of PFC, humans are less sensitive than rats and mice. Monkeys with their lower sensitivity would be a better model for humans in this respect [3]. Rodent data, in contrast, could lead to more cautious human health-based criteria following a procedure analogous to that of the German Drinking Water Ordinance.

Because of the lack of other adequate data, HRIV are based on criteria of evidence (e.g. genotoxic yes or no) and background knowledge about their level [2]. They constitute semi-quantitative assessment results that contain greater uncertainties, but generally are to be considered as protective. Therefore, HRIV are not derived for generic scenarios that are subject to measures or actions, but for single cases, whose special character, basic parameters, and possibly further appraisal factors have to be taken into account. In the present context, HRIV are utilized as criteria for the plausibility from a human health perspective of ecotoxicologically based GSF. That is why in assessing the occurrence of several PFC, HRIV should not be included in the summation. Since HRIV are based on insufficient data, they are of a precautionary nature and can, therefore, not be used as significance thresholds for groundwater.

An Italian EQS working group [71–74] derived “groundwater EQS” for PFPeA (3 µg/L), PFHxA (1 µg/L), and PFBS (3 µg/L), which are based on the German HRIV [8] and can, therefore, not serve as significance thresholds. Similarly, the Italian groundwater EQS for PFOA (0.1 µg/L; Italian EQS Working Group, [72, 75]) was based on secondary poisoning, a scenario excluded in the present work (cf. “Unaccounted-for scenarios” section). And finally, the Italian EQS Working Group [72, 76] proposed a human health-based groundwater EQS of 7 µg/L for PFBA according to Lud et al. [16], whereas the drinking water guide value for this substance was updated to 10 µg/L, because recent data suggested that a higher allocation to drinking water would probably be more realistic (pp 4–5 in [38]).

## Conclusion

The GFS values recommended for the assessment of groundwater contaminated by PFC and for supplementing the LAWA-list [26] are shown in Tables 4 and 5. These values serve as criteria for the decision whether actions to protect the quality of groundwater or remediate polluted groundwater are necessary. They were accepted by the German Drinking Water Commission in September 2016 [38, 67] and by the German States’ Soil Consortium (LABO) in December 2017. Their publication by the German States’ Water Consortium (LAWA) is expected in 2018 [17]. The GFS values presented here will then replace previous regulations.

## Outlook

After the designation of 13 priority-substances, the derivation of GFS values was possible only for seven of these. For the remaining substances mostly human health data were missing; three of them also lacked data for derivation of an ecotoxicological PNEC [17]. It would be desirable to close these gaps in the foreseeable future through additional appropriate tests or scientific studies. Besides the perfluorinated substances partially fluorinated compounds are increasingly introduced by industry, including analogous alkanoic and alkanesulfonic acids as well as classes, in which the carboxylic and sulfonic acid group is substituted by a different polar functional group, for instance polyfluorinated alcohols (so-called telomer alcohols), acrylates, methacrylates, sulfonamides, phosphates, phosphonates, phosphines, stearates or silanes, partially combined or linked by ether-bonds [77–80]. Admittedly, these substances are precursors, which are transformed either metabolically or in the abiotic environment to (oftentimes short chain) perfluorinated alkanoic or alkylsulfonic acids [81, 82].

H_4_PFOS is degraded by very slow desulfonation and subsequent oxidation predominantly to PFPeA and PFHxA [83]. Most of these compounds are less accumulating, but still, the parent compounds or the transformation products are hardly less persistent than the perfluorinated carboxylic and sulfonic acids, and have already been detected in surface waters, sediments and in groundwater [84]. Examples in Germany are ADONA^14^ or HFPO-DA^15^ [85–87]. At present, the limited analytical possibilities and the availability of analytical standards dictate, if and which of these chemical substitutes can be measured [81, 87].

The GFS values will retain their importance, even if the direct application of these substances should be definitely terminated. Beyond that, it is foreseeable that the measurement and assessment of additional perfluorinated and polyfluorinated chemicals will still be necessary in the future.

## Abbreviations

ADI: acceptable daily intake for lifelong exposure: threshold dose for contaminants with a certain benefit, e.g. for food additives
ADONA: ammonium-4,8-dioxa-3H-perfluorononanoate
CAS: chemical Abstracts Service, the CAS-No. is an international standard for the identification of chemical substances
EQS: environmental quality standard
GFS: *Geringfügigkeitsschwelle*—significance threshold for the assessment of contaminated groundwater
GOW: G*esundheitlicher Orientierungswert*—health-related indication value—HRIV [1, 2]
HFPO-DA: 2,3,3,3-Tetrafluor-2-(1,1,2,2,3,3,3-heptafluorpropoxy)propanoic acid
HRIV: health-related indication value; *gesundheitlicher Orientierungswert*—*GOW* [1, 2]
LABO: Bund/Länder-Arbeitsgemeinschaft Boden—German States’ Soil Consortium
LAWA: Bund/Länder-Arbeitsgemeinschaft Wasser—German States’ Water Consortium
LHL: Landesbetrieb Hessisches Landeslabor—Hessian laboratory management agency [Hessian State Laboratory]
LOQ: limit of quantitation
LW: drinking water guide value LW [µg/L] = TDI [µg/(kg day)] · 70 kg · (2 [L/day])^−1^ · 0.1
MRL: minimum risk level (ATSDR [3])
NOAEL: no observed adverse effect level
NOEC: no observed effect concentration
PFAS: per- and polyfluoroalkylated substances
PFC: per- and polyfluorinated compounds. PFC are also designated as per- and polyfluoroalkylated substances (PFAS) in the technical literature
PNEC: predicted no effect level
PPARα: peroxisome proliferatior-activated receptor α
SVHC: substance of very high concern
TDI: tolerable daily intake for lifelong exposure, threshold dose for contaminants with no benefit

## Footnotes

1 PFC are also designated as per- and polyfluoroalkylated substances (PFAS) in the technical literature.

2 TDI = Tolerable daily intake for lifelong exposure, threshold dose for contaminants with no benefit.

3 LHL = Landesbetrieb Hessisches Landeslabor.

4 ISO/CD 21675: Water quality—Determination of polyfluorinated alkyl substances (PFAS) in water-Method using solid phase extraction and liquid chromatography–tandem mass spectrometry (LC–MS/MS).

5 This also applies to food and animal feed.

6 In the regulations for marketing of fertilizer, soil additives, culture substrates and plant-growth stimulants [21] a reference value of 100 µg/kg dry weight was formalized for the cumulative concentration of PFOA and PFOS.

7 CAS = Chemical Abstracts Service, the CAS No. is an international standard for the identification of chemical substances.

8 NOAEL: no observed adverse effect level.

9 PNEC = predicted no effect concentration.

10 NOEC = no observed effect concentration.

11 ADI = acceptable daily intake for lifelong exposure: threshold dose for contaminants with a certain benefit, e.g. for food additives.

12 The maximum tolerable concentration in drinking water is designated as drinking water guide value (LW). LW [µg/L] = TDI [µg/(kg day)] · 70 kg · (2 [L/day])^−1^ · 0.1.

13 HRIV: precautionary health related indication value—*Vorsorge-orientierter gesundheitlicher Orientierungswert—GOW* [1, 2], cf. sections “Substances insufficiently characterized” and “Uncertainty analysis”.

14 ADONA = ammonium-4,8-dioxa-3H-perfluorononanoate.

15 HFPO-DA = 2,3,3,3-Tetrafluor-2-(1,1,2,2,3,3,3-heptafluorpropoxy)propanoic acid.
